# Chiral Glass Formation by Dipeptide Salts

**DOI:** 10.1021/acs.biomac.5c02634

**Published:** 2026-02-13

**Authors:** Valeria Castelletto, Ian W Hamley

**Affiliations:** School of Chemistry, Food Biosciences and Pharmacy, 6816 University of Reading, Whiteknights, Reading RG6 6AD, U.K.

## Abstract

A simple dipeptide WR (tryptophan–arginine) in the form of salts with organic acids tartaric acid or crotonic acid is shown to form glasses through a benign preparation route by evaporation of aqueous solution. The glasses have a remarkable range of properties including moldability, high transparency across a broad range of wavelengths, and fluorescence. The glasses show self-healing and adhesive properties, and have accessible glass transition temperatures. The glasses are shown to be amorphous via small-angle and wide-angle X-ray scattering (SAXS/WAXS) and scanning electron microscopy (SEM). Remarkably, the glasses are found to have a chiral structure, as shown by circular dichroism (CD) spectroscopy. Investigation of glass precursor dipeptide salt solutions shows that the glasses form from an initial unordered solution containing chiral peptide molecules. The diverse properties of the dipeptide glass materials points to a wide range of potential future applications.

## Graphical abstract

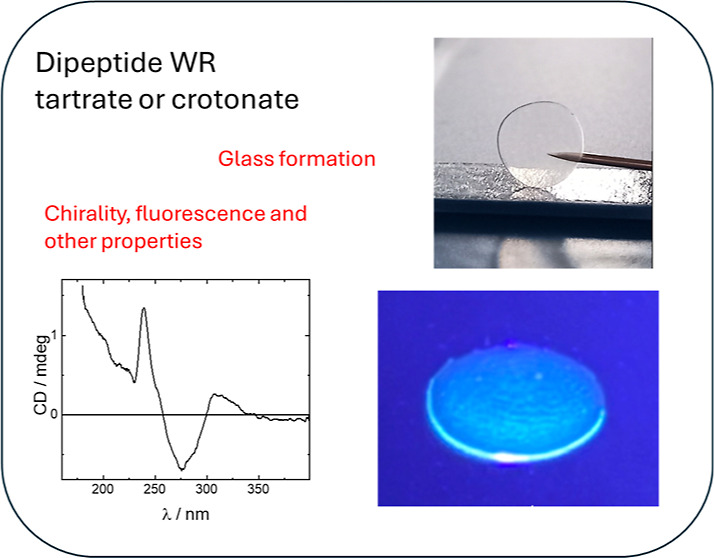

## Introduction

Glasses have been known to humanity for thousands of years, primarily in the form of silicates and other inorganic oxides and carbonates used in windows, bottles and optical applications. More recently glassy polymers have been developed for applications in containers or in eyewear. Glasses are amorphous solids, often considered to have a liquid-like structure with considerably reduced molecular dynamics.

Peptides are present in nature as chains of amino acids with a range of biological functions as hormones, in host defense, signaling, and others. Peptide sequences can be designed to target many other applications and can be routinely synthesized using automated methods such as solid phase peptide synthesis (SPPS). Peptides can form well-known secondary structures such as α-helices and β-sheets.^1–3^ They can crystallize or, depending on sequence, they can self-assemble or aggregate in solution as in the case of “amyloid” peptides forming β-sheet fibrils for example^4–6^ or due to surfactant-like structure^7–10^ or from specific intermolecular interactions such as π-stacking.^11–13^ Many peptides can also form gels in water or organic solvents.^14–16^ However, there are few reported examples of peptide glasses although these materials hold promise due to the biocompatibility and/or biofunctionality of peptides, and their easy production from renewable resources. The distinct processes of self-assembly of peptides forming ordered nanostructures or disordered structures including droplets from liquid–liquid phase separation (LLPS) or glasses have recently been discussed.^17^

The tyrosine tripeptide (YYY) has been shown, upon dehydration of an aqueous solution, to form a glass with a remarkable variety of properties including self-healing, strong adhesion and a wide spectral range of transparency.^18^ The glass formation was ascribed to the formation of an extensive hydrogen bonding network of the tyrosine hydroxyl groups (as well as backbone amide groups) with water molecules. In fact a wide range of amino acids, short peptides and even longer peptides and peptide conjugates complexed with organic acids have been shown to form glasses with a remarkable diversity of properties including tunable refractive index, ability to be 3D printed, drawn into fibers or molded and with programmable recyclability and/or humidity responsiveness.^19^ *N*-terminally modified (with Fmoc, 9-fluorenylmethoxycarbonyl or Cbz, benzyloxycarbonyl) peptides or amino acids (without organic salts) can also form glasses through a melt-quench process.^20^ In another recent study, Li and co-workers have reported glasses formed by basic amino acids including histidine with organic acids such as tartaric acid (or acidic amino acids with organic bases).^21^ In the case of histidine with tartaric acid, either a glass or a crystal can be formed depending on the evaporation rate of a solution of the amino acid/tartaric acid solution. The glass formation was ascribed to the formation of an extensive hydrogen bonding network of histidine and tartaric acid molecules (the packing was modeled based on the crystal structure). The resulting glasses demonstrate a range of interesting properties including high transparency over a broad spectral range and multicolor fluorescence when excited with light of different wavelength. Doping the glass with a phosphorescent compound leads to extended time phosphorescence (with long afterglow).^21^ Examples of previous work on glass-forming peptides are summarized in Supporting Information Table S1.

We have recently been studying model sequenced peptides containing tryptophan and arginine. These show interesting self-assembly behavior and functionality due to combination of the aromatic tryptophan residue along with the basic arginine residue. Self-coacervation (liquid–liquid phase separation, LLPS) occurs for W_2_R_2_ and W_3_R_3_ peptides in basic solutions, arising from π–π and cation–π interactions of the tryptophan residues.^22^ The complex coacervation of the shorter peptide WR with ATP was also noted. We further investigated the influence of sequence length in (WR)_*n*_ peptides with *n* = 2–5 on self-assembly/aggregation.^23^ Whereas (WR)_2_ and (WR)_3_ can form coacervate droplets due to LLPS, depending on pH, the longer (WR)_4_ and (WR)_5_ peptides form extended β-sheet structures (twisted nanotapes). The longer peptides also form hydrogels comprising β-sheet fibrillar structure at higher concentration.^23^

Here we report on glass formation by a dipeptide WR. We show that when slowly evaporated from peptide salts in the presence of hydrogen-bonding capable organic acids, tartaric acid or crotonic acid, it is possible to prepare glasses. The glasses have a great range of interesting properties of which the chirality of the glass is particularly notable. Other features include moldability, high transparency (high UV/vis transmission), fluorescence, adhesion to form load-bearing joints, and self-healing. Notably, the glasses show accessible glass transition temperatures near body temperature that may be useful for future activities in biomedicine. We also show that the glasses are amorphous, using cryo-TEM and SAXS/WAXS along with FTIR spectroscopy to probe conformation. The structure of the precursor solutions from which the glasses was prepared was also determined via SAXS/WAXS, CD and FTIR and this shows that the glasses are formed from peptide in unaggregated form.

## Materials and Methods

### Materials

Peptide salts (with tartaric acid or crotonic acid) of WR (NH_2_-tryptophan-arginine–OH) were obtained from Peptide Synthetics (Peptide Protein Research, Farnham, UK) with >95% purity as confirmed by RP-HPLC. The peptide molar mass by ESI-MS is 360.4 g mol^–1^ (360.4 g mol^–1^ expected). Tartaric acid and crotonic acid were obtained from Merck Sigma-Aldrich (Gillingham, UK). Scheme 1 shows the molecular structures of WR and the organic acids.

**Scheme 1:**
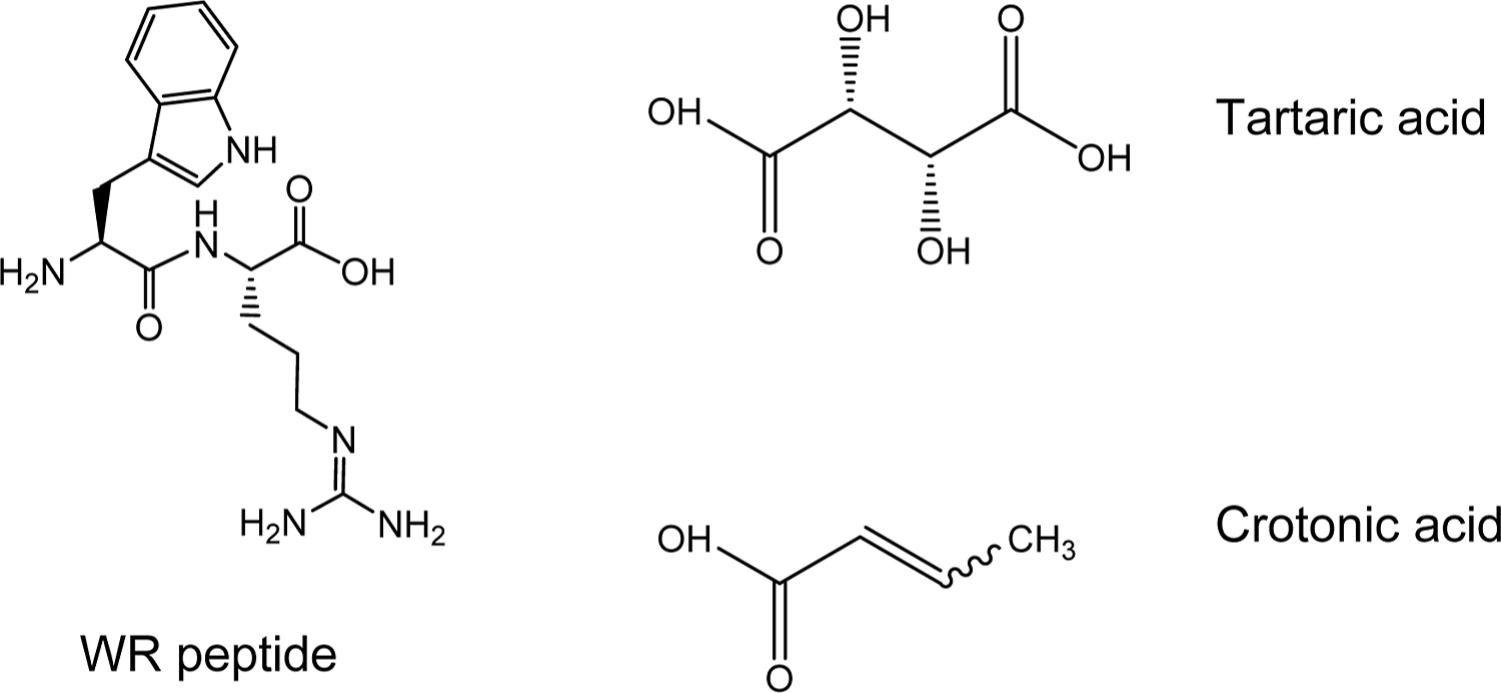
Molecular Structure of WR and Tartaric Acid and Crotonic Acid

### Glass Preparation

Glasses were prepared using 10 wt % WR crotonate or 10 wt % WR tartrate solutions in water. Both solutions, prepared by weighing convenient amounts of peptide and water were homogenized by 20 min of alternated ultrasound and vigorous vortex cycles. Thereafter, a 20 or 30 μL drop of the final solution was placed on the corresponding substrate, as detailed below, and left to dry for 24 h inside a sealed desiccator loaded with silica gel.

It was found that a nonadhesive silicone rubber substrate produced flat glass disks for both WR crotonate and WR tartrate. However, flat glass disks of WR tartrate were more efficiently produced by using a self-adhesive silicone rubber tape substrate. Interestingly, the self-adhesive silicone rubber tape surface produced concave-textured disks for WR crotonate; a result correlated to the surface tension of the drop placed on the substrate. Glass disks were detached from the silicone surface by careful manual bending of the substrate.

A separate set of glasses, for healing experiments, was prepared using a microscope slide as a substrate. This produced multiple cracks on the peptide glass surface, which were healed by humidified thermal healing experiments detailed below.

### Scanning Electron Microscopy

Glass disks were placed on a stub covered with a carbon tab (Agar Scientific, U.K.) and then coated with gold. A FEI Quanta FEG 600 environmental scanning electron microscope (SEM) in high vacuum mode (5 kV high tension) was used to record SEM images.

### Differential Scanning Calorimetry

Experiments were performed using a TA Instruments Multi-Sample X3 DSC instrument. For the experiments, each glass sample was loaded in a TA Instruments standard hermetic pan. A ramp rate of 10 °C/min was used for all experiments. The temperature was first decreased from 40 °C to −40 °C. The sample was left to equilibrate at −40 °C for 10 min. A temperature ramp −40 °C → 120 °C was started following equilibration at −40 °C. This was followed by a final temperature ramp 120 °C → −40 °C.

### Thermogravimetric Analysis

Experiments were performed using a TA Instruments TGA Q50. Both glass samples were loaded in a TA Instruments standard hermetic pan. Experiments were run using a 10 °C/min T-ramp. The first derivative of the experimental data was calculated using Origin-Lab software.

### Fourier Transform Infrared Spectroscopy

Experiments were performed using a PerkinElmer Spectrum 100 FTIR-ATR instrument. A portion of glass disk was pressed against the crystal using the high-pressure clamp accessory while measuring the FTIR-ATR spectra of the glasses. Precursor solutions containing 10 wt % WR with salt were prepared in D_2_O; a drop of the solution was placed on the crystal to measure the spectrum.

### UV–Vis Absorption

Spectra were recorded using a Varian Cary 300 Bio UV–vis spectrometer. A 0.1 mm light path parallel plaque quartz cell, consisting of a dented plaque and a flat plaque, was used for the experiments. The well of one was filled with 10 wt % precursor peptide salt solution and left to dry for 24 h in a sealed desiccator loaded with silica gel. After drying, the precursor solution turned into a glass. Following glass formation, a flat plaque was placed on top of the plaque holding the glass and the sandwiched cell was used for UV–vis experiments.

### Circular Dichroism Spectroscopy

Far-UV CD spectra were collected using a Chirascan spectropolarimeter (Applied Photophysics, Leatherhead, UK). Spectra were recorded from 180 to 400 nm. Samples were mounted in a quartz cell with detachable windows, with 0.01 mm path length. The CD signal from the samples was corrected by water background subtraction. The CD signal was smoothed using the Chirascan Software for data analysis. The residue of the calculation was chosen to oscillate around the average, to avoid artifacts in the smoothed curve. For solutions, CD data, measured in mdeg, was normalized to molar ellipticity using the molar concentration of the sample and the cell path length. For the solid glass samples, WR salt precursor solution was dried between 0.01 mm quartz parallel plaques (one with a shallow well to hold solution) and data is presented in mdeg.

### Fluorescence Spectroscopy

Experiments were performed using a Varian Cary Eclipse spectrofluorimeter. Fluorescence emission experiments were measured from 300 to 500 nm using an excitation wavelength λ_ex_ = 280 nm. Peptide solutions were placed inside a quartz cell with 10.0 × 5.0 mm^2^ internal cross section. For the solid glass sample, WR crotonate or tartrate precursor solutions were dried between 0.01 mm quartz parallel plaques.

### Small-Angle X-ray Scattering Experiments and Wide-angle Scattering

SAXS/WAXS experiments were performed on beamline B21 ^24^ at Diamond (Didcot, UK). Sample solutions were loaded into the 96-well plate of an EMBL BioSAXS robot and then injected via an automated sample exchanger into a quartz capillary (1.8 mm internal diameter) in the X-ray beam. The quartz capillary was enclosed in a vacuum chamber, to avoid parasitic scattering. After the sample was injected into the capillary and reached the X-ray beam, the flow was stopped during the SAXS data acquisition. Glass samples were mounted in custom built polycarbonate multipurpose sample holders,^25^ held with Superio (Mitsubishi Chemical) UT F-type poly­(ether imide) film (7 μm thickness) (which provides very low SAXS background) which were inserted into the sample chamber in the beamline.

Beamline B21 operates with a fixed camera length (3.9 m) and fixed energy (12.4 keV). The SAXS images were captured using a PILATUS 2M detector. The WAXS data was acquired using a Dectris EIGER 2 R 1M M detector, and the *q*-axis was calibrated using the diffraction spectrum of silver behenate and the SAXS intensity was normalized using the signal of water. Data processing was performed using dedicated beamline software ScÅtter.

### X-ray Diffraction

Measurements were performed on tartaric acid crystals mounted on an Oxford Diffraction Gemini Ultra instrument. The sample–detector distance was 60 mm. The X-ray wavelength λ = 1.54 Å was used to calculate the scattering vector *q* = 4π sin θ/λ (2θ: scattering angle). The detector was a Sapphire CCD.

### Humidified Thermal Healing Experiments

A 20 μL drop of 10 wt % WR crotonate or tartrate in water was placed on the surface of a microscope slide and left to dry for 24 h inside a sealed desiccator loaded with silica gel. The glass formed on the microscope slide was cracked. An Olympus BX-41 optical microscope was used to take images. A few drops of water were placed around the peptide glass on the surface of the microscope slide. Thereafter, the microscope slide was enclosed inside two Petri dishes and placed in an oven at 75 °C for 9 min. Afterward the microscope slide was observed under the microscope to examine the healed surface of the peptide glass.

## Results

Glass formation was observed (Figure 1) for dipeptide WR salts after slow evaporation from aqueous solutions of the tartaric acid or crotonic acid (Scheme 1), which contain hydroxyl and/or carboxyl groups able to form hydrogen bonds with the amide units. The development of a network of hydrogen bonds is believed to underpin the formation of peptide glasses.^18,19,21^ Dipeptide WR is also able to interact with the salts through electrostatic interactions. The glasses can easily be molded as shown in Figure 1a,b which present images of disks formed from WR tartrate and crotonate. Supporting Information Figure S1 shows an additional image of a patterned glass molded from WR crotonate, with structural features arising from surface tension/adhesion to the surface. The structure of the glasses was examined by cryo-SEM and SAXS/WAXS. The representative cross-section cryo-SEM image in Figure 1c (additional images provided in Supporting Information Figure S2 for both WR salt glasses) shows an amorphous structure, i.e. no internal morphology could be discerned. The surfaces of the glasses are also smooth and featureless (Supporting Information Figure S3). The amorphous structure of the glass was also confirmed by SAXS/WAXS. The SAXS data in Figure 1d is featureless, with a featureless upturn in the scattered intensity at low *q* due to density fluctuations in the glass. The WAXS data (Figure 1d) for the crotonate glass is also featureless (same shape as the background curve) whereas for the tartrate glass there is a series of small Bragg reflections superposed on the amorphous scattering. This is assigned to the presence of small tartrate crystallites within the glass, as confirmed by comparison with a measured diffraction pattern of tartaric acid (Supporting Information Figure S4).

**Figure 1:**
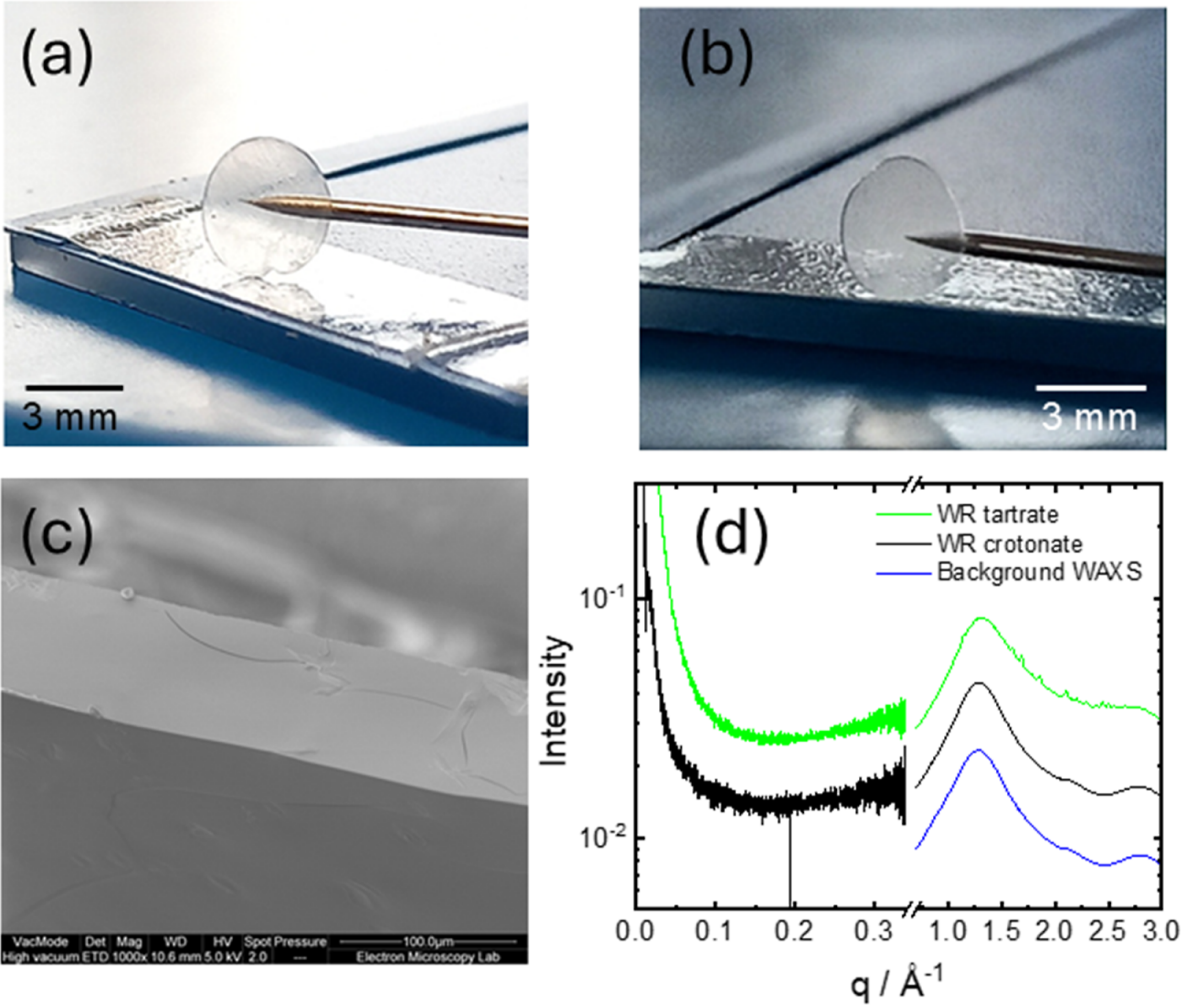
WR dipeptide glasses: molding and morphology. (a) Image of disk of WR tartrate prepared from a 10 wt % peptide solution, (b) image of disk of WR crotonate prepared from a 10 wt % peptide solution, (c) cross-section SEM image of WR tartrate glass, (d) SAXS/WAXS from WR glasses as indicated. The data has been scaled by multiplication of the intensity for ease of visualization.

SAXS data for aqueous salt solutions of WR shown in Supporting Information Figure S5 demonstrates that the peptide is present in monomeric form in the 10 wt % peptide salt precursor solutions from which glasses were prepared, with a characteristic plateau for intermediate *q* and smooth downturn at high *q*, features of SAXS scattering from monomers,^22,26^ with only a small upturn observed at low *q* due to irregular aggregation. The 1 wt % peptide solutions show scattering from monomeric unaggregated peptide (Supporting Information Figure S5) These measurements indicate that the glasses form from initial disordered, largely monomeric, solutions of the peptide salts.

The thermal properties of the glasses were analyzed. The glass transition temperature (*T*_g_) was first determined using differential scanning calorimetry (DSC) and the data obtained using these methods is shown in Figure 2. The glass transition is clearly defined for WR tartrate at 32.3 °C (Figure 2a) and for the crotonate glass at 35.6 °C. These accessible glass transition temperatures, close to body temperature may be beneficial for applications such as in vivo release of encapsulated cargo. Thermogravimetric analysis (TGA) was used to determine the thermal degradation of the glasses, which were found to be stable up to temperatures above 200 °C (Figure 2c,d) showing the high thermostability of the glasses. The first derivative analysis of the TGA thermograms provides the weight loss onset, maximum degradation rate and complete degradation temperatures (Supporting Information Figure S6).

**Figure 2:**
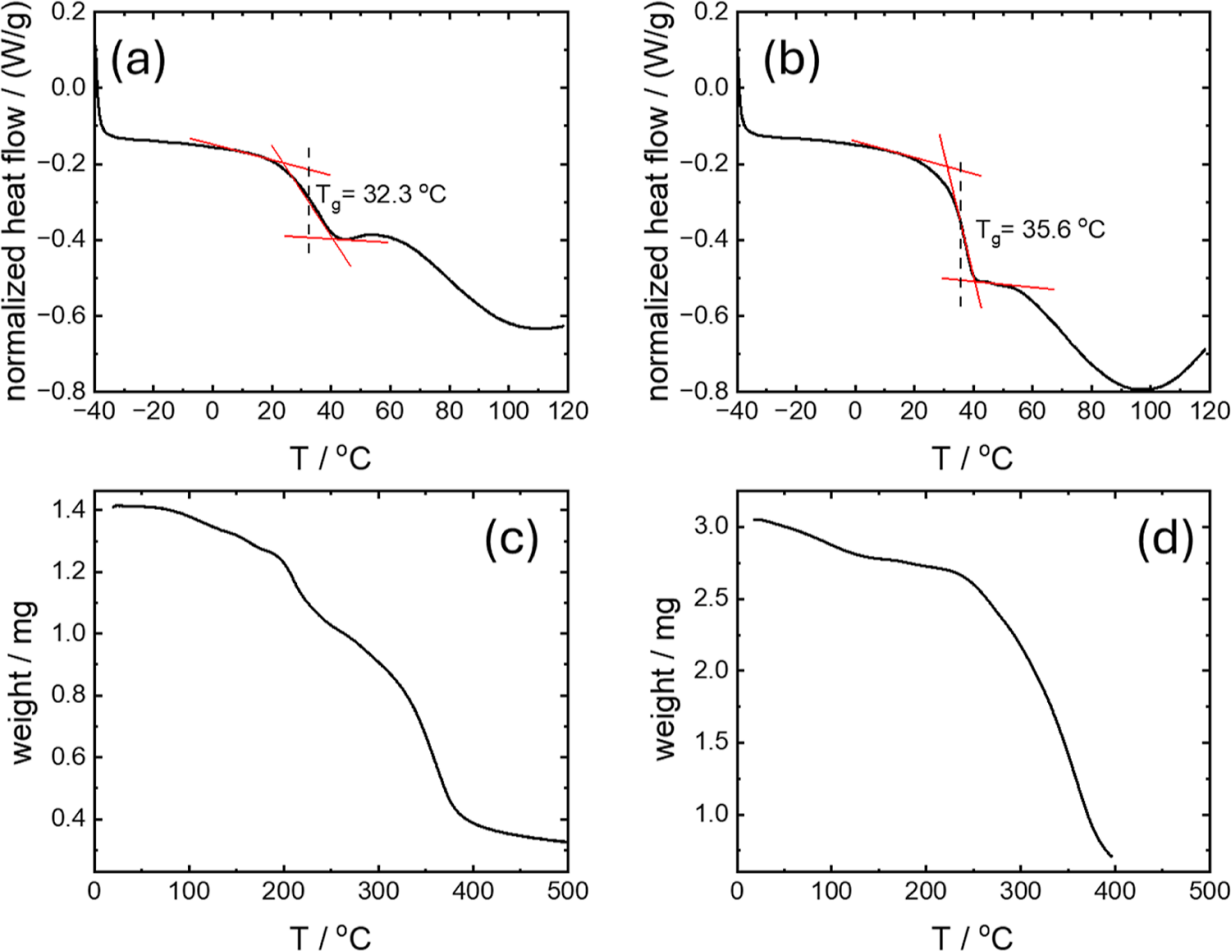
Thermal properties of glasses prepared from 10 wt % WR solution. (a) DSC heating scan for WR tartrate, (b) DSC heating scan for WR crotonate, (c) TGA scan for WR tartrate, (d) TGA scan for WR crotonate.

The glasses are amorphous as shown by cryo-SEM and SAXS/WAXS, and spectroscopic methods were also used to probe peptide ordering/conformation in the glassy state. Figure 3a shows FTIR spectra obtained for the glasses. The conformation of the peptide salts in the precursor solutions was also examined to shed light on the initial state prior to vitrification and the corresponding FTIR for precursor solutions is shown in Figure 3b. The FTIR spectra for the glasses show very distinct peaks compared to those for powders of tartaric acid^27,28^ or crotonic acid,^29^ which were measured for comparison. There are peaks for the WR tartrate at 1660 cm^–1^ due to turn structure^30,31^ and 1562 cm^–1^ and 1622 cm^–1^ due to Arg and Trp side chain deformation modes.^31,32^ The peaks at 1656 cm^–1^ and 1631 cm^–1^ for the WR crotonate may be associated with red-shifted peaks from crotonic acid^29^ or, since peaks in similar positions are observed for the tartrate (while the tartaric acid itself shows no peaks in this wavenumber range), they are more likely due to peptide turn structure or side chain deformation modes. The peaks at 1567 cm^–1^ and 1524 cm^–1^ are assigned to Trp side chain modes.^31,32^ The FTIR spectra for the peptide salt precursor solutions are shown along with that for the trifluoroacetic acid (TFA) salt (which does not show glass formation) in Figure 3b. The spectra for WR tartrate and WR crotonate show a strong peak in the amide I region at 1660–1664 cm^–1^ due to hydrogen bonded amide groups, possibly with significant turn structure.^30,31^ The WR TFA salt shows a peak at 1672 cm^–1^ due to bound TFA ions.^33–35^ The spectra show a peak at 1598 cm^–1^ for all three salts (thus not a signal of glass formation) assigned to an arginine side chain mode.^31,32^ The peak at 1543 cm^–1^ observed for the crotonate salt suggests a specific interaction between crotonic acid and the WR peptide (it is not present for crotonic acid, Figure 3a nor for WR TFA solution) probably involving interaction of the crotonic acid with Arg and/or Trp side chains. There is also a featureless peak for each WR peptide salt solution due to hydrogen-bonded N–H stretch deformations at 3400 cm^–1^.^30,36^ The FTIR spectra for the glasses and precursor solutions both indicate that the peptide does not have an ordered conformation (β-sheet or α-helix) in either state. This is consistent with the amorphous nature of the glass.

**Figure 3:**
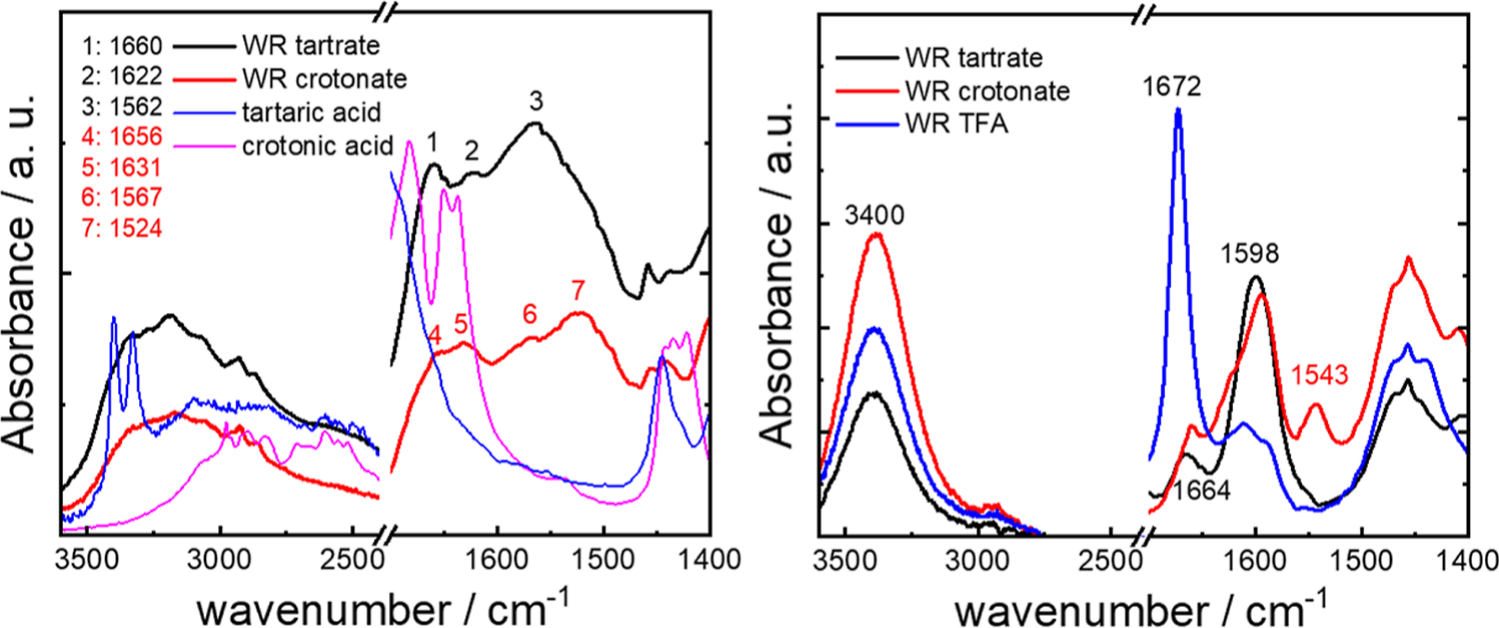
FTIR spectra highlighting amide A and amide I regions. (a) glasses with reference spectra for powders of the organic acids, (b) 10 wt % peptide salt precursor solutions and nonglass forming 10 wt % WR TFA (trifluoroacetic acid) solution.

The dipeptide glasses have a range of interesting optical properties. They are transparent as shown by the images in Figure 4a,b. The measured transmittance is above 75% in the wavelength range 300–800 nm for WR tartrate and nearly 100% for the crotonate salt in the same range (Figure 4c,d). The transmittances are reduced below λ = 300 nm due to the absorption features from the tryptophan residue, as evident from the absorption spectra in Figure 4c,d. As is apparent from the images in Figure 4e,f the glasses are fluorescent (an additional image of WR crotonate glass fluorescence is provided in Supporting Information Figure S7). The fluorescence spectra of the glasses are shown in Supporting Information Figure S8. The peak in the spectra results from the tryptophan fluorescence, the precursor solutions showing a fluorescence maximum near λ = 360 nm when excited at λ = 280 nm (Supporting Information Figure S9). Circular dichroism (CD) spectroscopy reveals that the peptide glasses are chiral, reflecting the chirality of the dipeptide. The spectra for both salts in Figure 4g features a positive maximum at λ = 239 nm (crotonate) or λ = 233 nm (tartrate) which is assigned as a red-shifted peak due to tryptophan absorbance, In addition each spectrum has a negative maximum around λ = 270–280 nm due to the Cotton effect from the local chiral environment. It should be noted that the magnitude of the CD signal was found to depend on the glass preparation conditions as well as the sample thickness.

**Figure 4:**
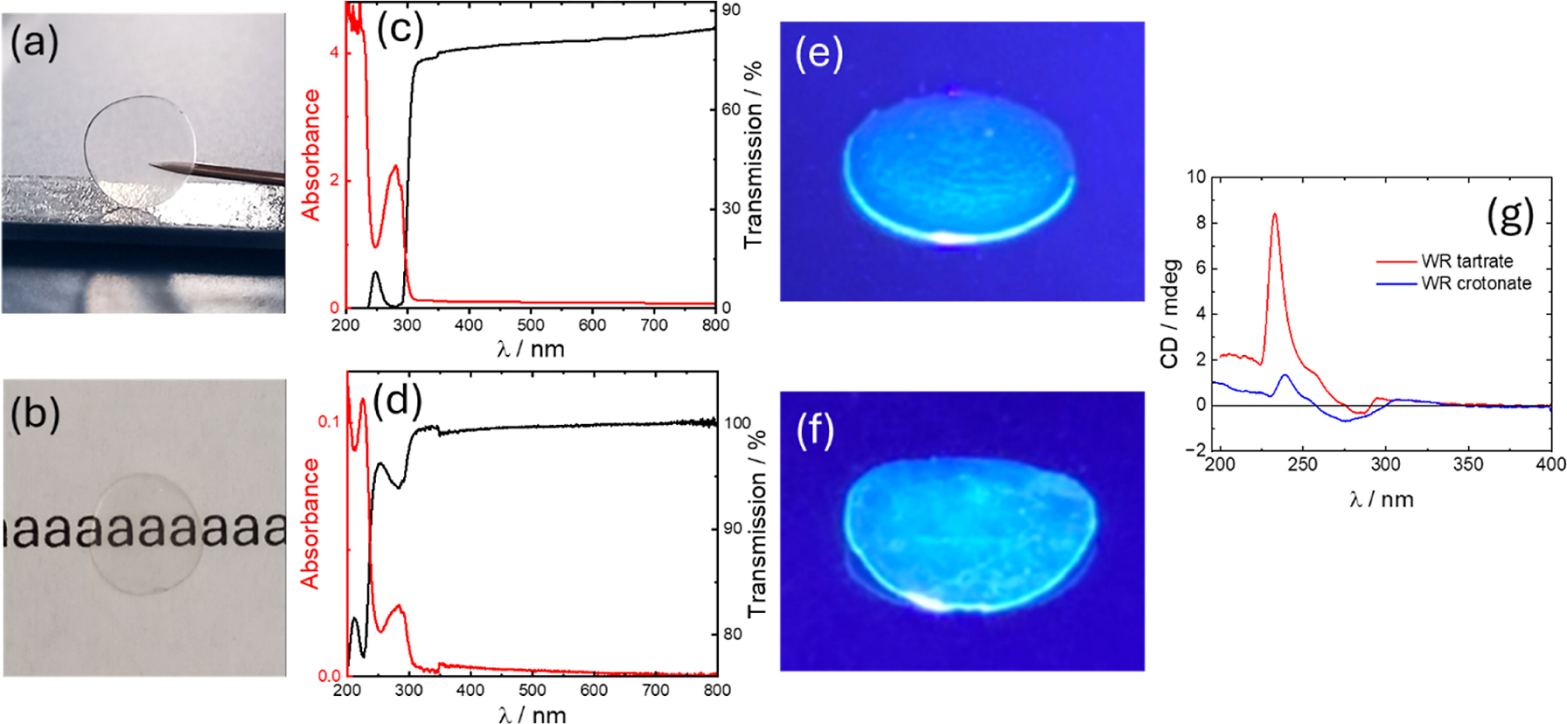
Optical properties. (a,b) Images of WR crotonate showing transparency. Measured UV/vis transmission/absorbance spectra for (c) WR tartrate, (d) WR crotonate. Images of fluorescent glasses upon illumination with 395 nm light (e) WR tartrate, (f) WR crotonate, (g) CD spectra of glasses.

CD spectroscopy was also used to probe peptide conformation in precursor solutions. The spectra are presented in Supporting Information Figure S10 and show contributions from the WR peptide and, in the case of chiral tartaric acid, the salt. The CD spectrum from l-tartaric acid with a minimum at 216 nm resembles that previously reported.^37^ Crotonic acid is nonchiral. The spectra for the WR peptide salts in solution have similar features to those in the spectra for the TFA (trifluoroacetate) salt,^22^ as shown in Supporting Information Figure S10, in particular a positive maximum a 225 nm due to the W residue, a negative minimum at 210 nm and a positive shoulder maximum at 198 nm. The lack of features from an ordered secondary structure is consistent with the FTIR spectroscopy data, and again confirms that the glass forms from a precursor solution comprising unaggregated peptide in a disordered conformation, although with features due to the chirality of the peptide with electronic transitions of the tryptophan indole group.

The glasses can be used to form adhesive bonds as exemplified in Figure 5a, in which a WR tartrate glass was formed in situ between two glass slides, which are then bonded. The bonded assembly can support significant weights, as illustrated by the 200 g load-bearing capacity in Figure 5b. It should be noted that the glasses presented here, prepared by a simple drying method from aqueous precursor solutions are water-soluble, therefore the adhesive bonds illustrated in Figure 5a,b can be dissolved by water, i.e. the peptide-based system serves as washable adhesive, with potential for easy-clean and no-mess applications. Considering the glass transition temperatures reported above, the adhesive bonds can also be thermally broken at modest temperature (for practical application the *T*_g_ should be tuned by adjustment of the initial peptide and/or salt concentration and/or by blending with other components^38^). The glasses were also observed to exhibit self-healing when exposed to water vapor in a Petri dish heated to 75 °C, as illustrated by the image of an initially cracked WR crotonate glass in Figure 5c, which self-heals such that the appearance of cracks is eliminated within 10 min (Figure 5d). An additional image showing healing of a glass is provided in Supporting Information Figure S11. Full healing was not observed in a control experiment performed without a humid atmosphere. Self-healing was also observed for a WR tartrate glass as shown by the images presented in Supporting Information Figure S12, healing occurring within 4 min. The images show that the cracks appear to heal via intermediate arrays of drop-like structures which form within 1–2 min and are then annealed out over a few more minutes. Higher magnification optical microscopy images shown in Supporting Information Figure S13 show that the self-healing of WR tartrate is accompanied by partial crystallization of the tartaric acid, consistent with the WAXS data discussed above (shown in Figure 1d and XRD data in Supporting Information Figure S4). The images suggest that this crystallization (formation of tartaric acid crystallites within the glass) is complete within 20 min, as no further development was observed after longer times. This was confirmed by images obtained after the thermal annealing for samples at room temperature shown in Supporting Information Figure S14.

**Figure 5:**
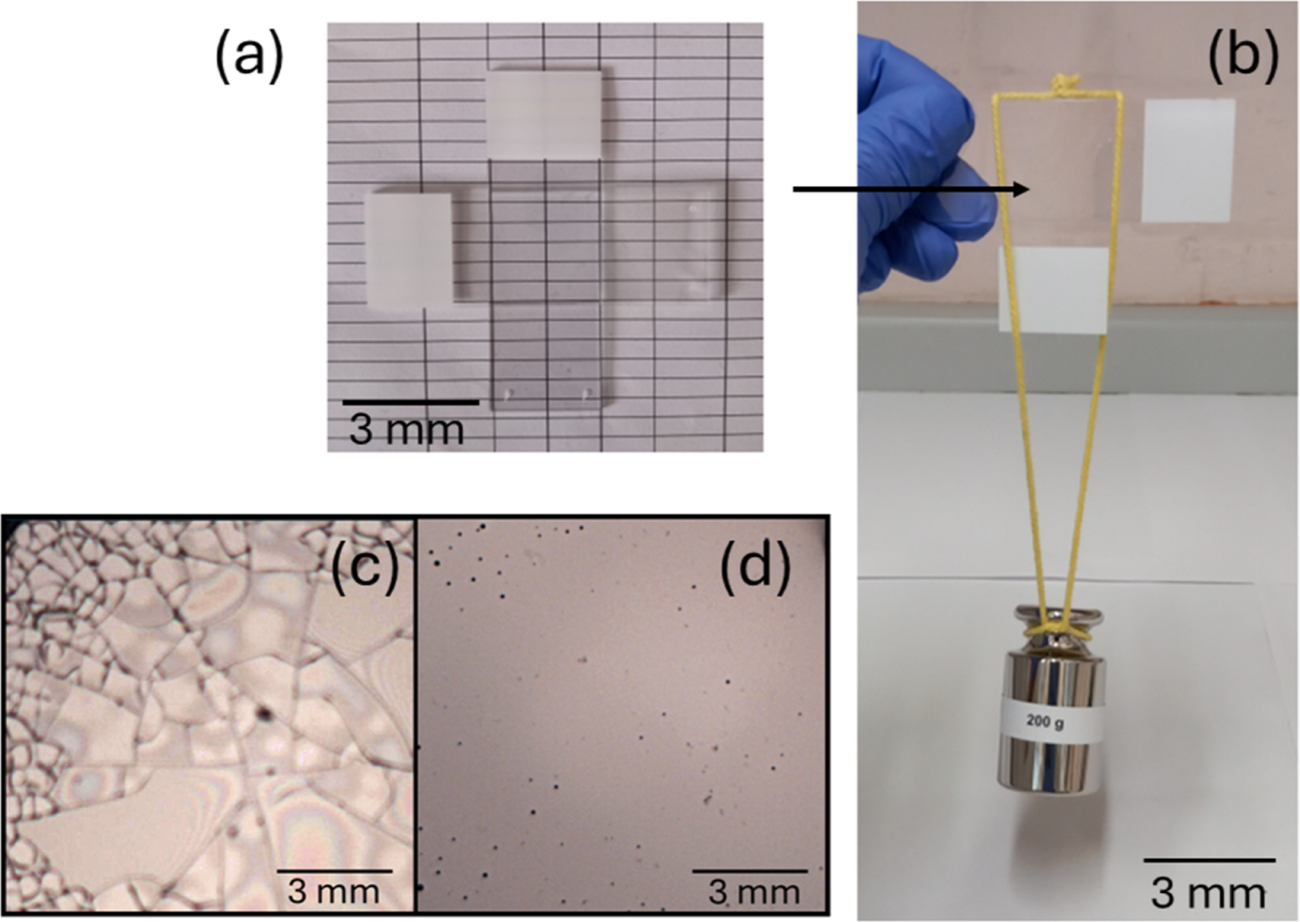
Adhesion and load-bearing capacity of WR tartrate glass. (a) An adhesive bond is formed between two glass slides and (b) the assembly is used to bear a load of at least 200 g (right). (c,d) Optical microscopy images showing self-healing under humid conditions of a cracked glass of WR crotonate as prepared (c), and after 10 min self-healing (d).

A further interesting property of the glasses that was noted was their surface static charge, which leads to the property that glass particles are repelled from similarly charged surfaces due to electrostatic phenomena. A movie showing this effect is included as Supporting Information Movie S1. The charge on the particles must arise from a net charge on the peptide/salt complexes.

## Conclusions

In summary, salts of dipeptide WR with the organic acids can be used to produce glasses under benign conditions from aqueous solution based on biocompatible and renewable materials. The glasses are amorphous as revealed by SAXS/WAXS and SEM (with some crystallinity for the tartrate glasses). The glasses are formed from amorphous peptide salt precursor solutions and the dipeptide chirality is retained in the glass. The amorphous structure revealed by SAXS/WAXS and SEM indicates the absence of supramolecular chiral structures and suggests the chirality of the glasses arises from the local vitrified environment of the peptide molecules. The formation of glasses is ascribed in part to the development of a hydrogen bonding network facilitated by the presence of carboxyl and/or hydroxyl groups (crotonic acid lacks hydroxyl groups) on the organic salts and amine and amide group H-bond donors and carboxyl group donors/acceptors on the peptides.^21^ Electrostatic interactions are also likely to play a significant role in the stabilization of molecular aggregates in the glass, in particular involving the carboxylic acid groups on the organic acids and the peptide *N*-terminus and arginine residue. The effects of H-bonding and electrostatic interactions are manifested by changes in the FTIR spectra. As noted in our previous studies,^22,23^ cation–π interactions are important in the aggregation of the WR peptide itself in the absence of the organic salts (due to the presence of cationic arginine and aromatic tryptophan), and these self-interactions are likely to be influenced by distinct electrostatic and hydrogen bonding interactions in the mixed salt system.

The WR crotonate glass shows very high transmittance of light in the 300–800 nm wavelength range. The WR tartrate glass shows lower transmittance, and apparently higher peak molar ellipticity, although it should be considered that both the tartaric acid and the peptide are chiral. The WR crotonate also shows a glass transition temperature *T*_g_ near body temperature, potentially useful for future biomedical applications for instance in vivo degradation and release of encapsulated cargo. A further advantage of accessible *T*_g_ and humidity responsiveness may be for read/write data storage applications and to detect environmental conditions (temperature, humidity)^19^ detection, e.g. for food packaging. The glass transition temperature and other properties (solubility) can be tuned by blending or incorporation of other materials, for example metal ions^39^ which can be used to create much more resilient glasses, where this is needed for specific applications. It will also be interesting to examine in the future peptide glasses formed from other hydrogen-bond capable organic salts.

The dipeptide glasses exhibit diverse properties including their processability (here molding is demonstrated but other methods such as fiber drawing and 3D printing will be examined in future studies) and optical properties, notably fluorescence upon exposure to visible 385 nm light, which may be valuable for future development of fluorescence tags or labeling systems. The glasses can serve as water-based adhesives, providing high strength bonds that are nevertheless water-soluble, suggesting future applications in washable adhesives. The glasses show self-healing properties in humid atmospheres, again under benign conditions in contrast to high temperature processing required for many self-healing systems. Glasses are considered as nonequilibrium states, although in many cases very long-lived, and it will be interesting in future to examine the long-term stability of the glasses, which may depend on the salt. Here we present evidence that the tartaric acid in the WR glass crystallizes within 10–20 min, whereas this was not observed for the crotonate salt. Although chiral glasses are known for inorganic materials, especially superconductors, and are modeled as three-dimensional Heisenberg spin glasses, there are few reports to date on peptide-based chiral glasses^40,41^ and the simple WR dipeptide salts are notable new examples with a combination of other intriguing properties.

## Supplementary Material





## Notes

Additional image of molded glass, additional cryo-SEM images, WAXS and XRD data, SAXS data for precursor solutions, TGA first derivative plots, additional images of fluorescent glasses, fluorescence spectra, CD spectra from precursor solutions, additional images showing self-healing (PDF)
**Movie S1** showing electrostatic repulsion of tartaric acid glass fragment from a rubbed silicone tape (blue) (MP4)
