# Supplementary material for: Chiral Glass Formation by Dipeptide Salts
Source: Biomacromolecules. 2026 Feb 13;27(3):2251–9. doi: 10.1021/acs.biomac.5c02634 (PMC12977033; doi:10.1021/acs.biomac.5c02634)
Supplement: Supplementary file 1 [file bm5c02634_si_001.pdf]

Supporting Information

Chiral Glass Formation by Dipeptide Salts

Valeria Castelletto,[1] Ian W Hamley[1,*]

[1] *School of Chemistry, Food Biosciences and Pharmacy, University of Reading, Whiteknights, Reading RG6 6AD, U.K.*

* Author for correspondence. I.W.Hamley@reading.ac.uk

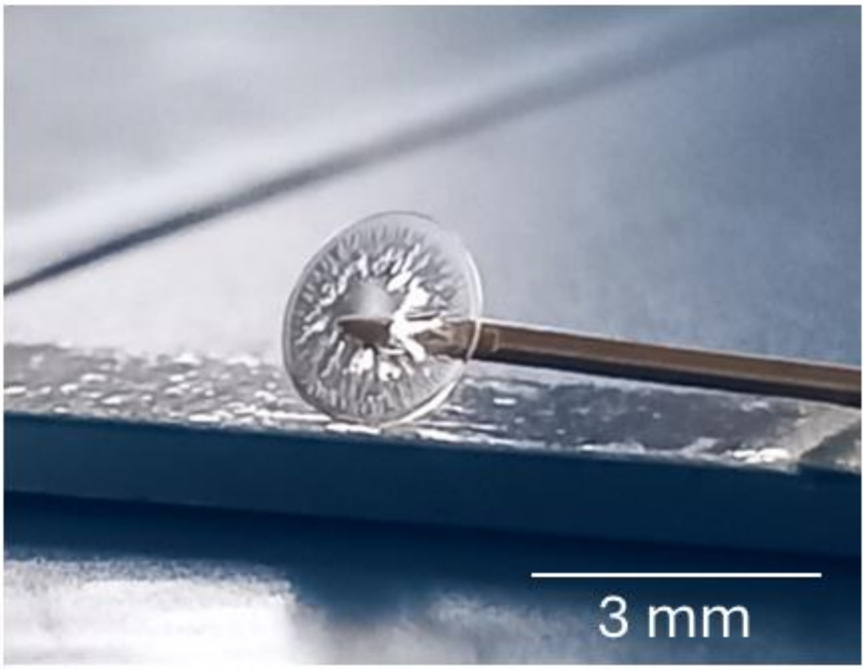

SI Fig.S1. Patterned glass of moulded WR crotonate.

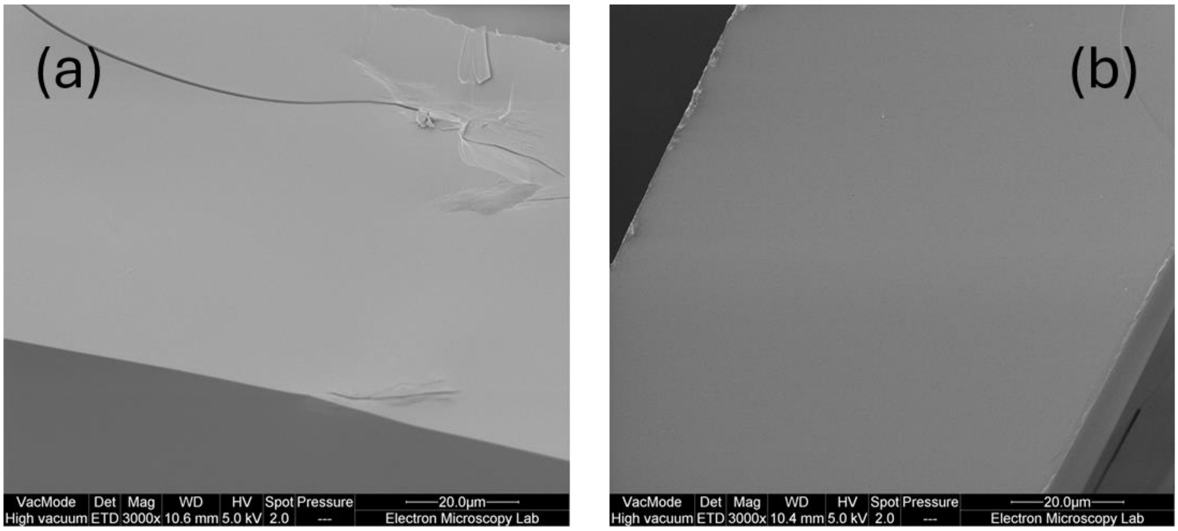

SI Fig.S2. Cryo-SEM cross-section images for glasses, (a) WR tartrate, (b) WR crotonate.

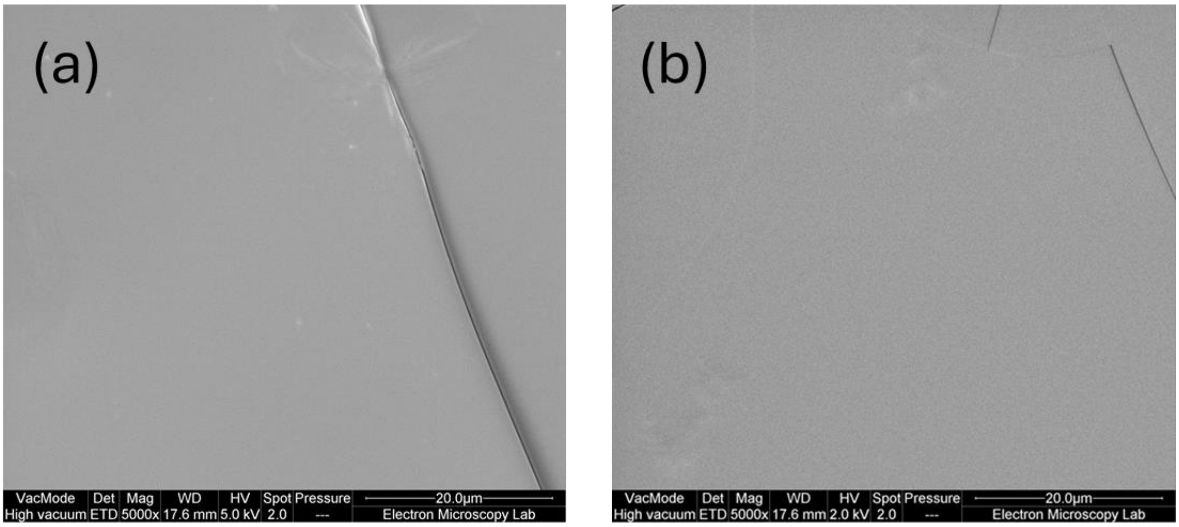

SI Fig.S3. Cryo-SEM images of glass surfaces, (a) WR tartrate, (b) WR crotonate. The cracks visible on the right-hand side of the images are used as focussing references for the featureless surface.

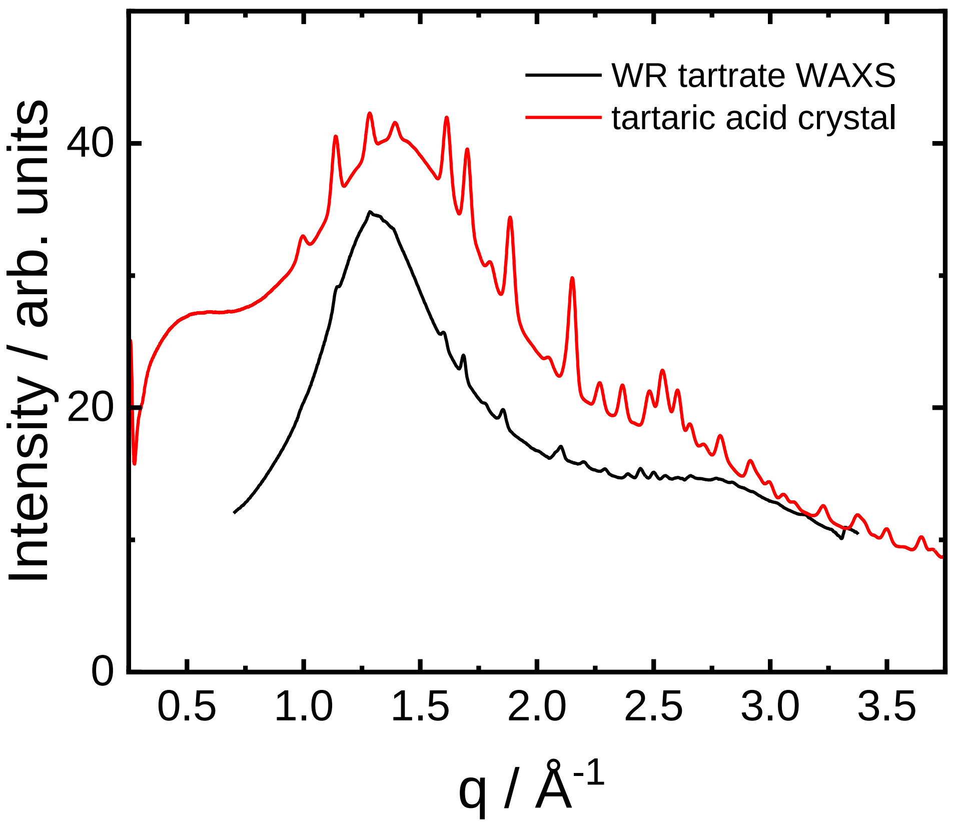

SI Fig.S4. Comparison of WAXS data for WR tartrate (same as Fig.1d) with measured diffraction pattern for tartaric acid crystal.

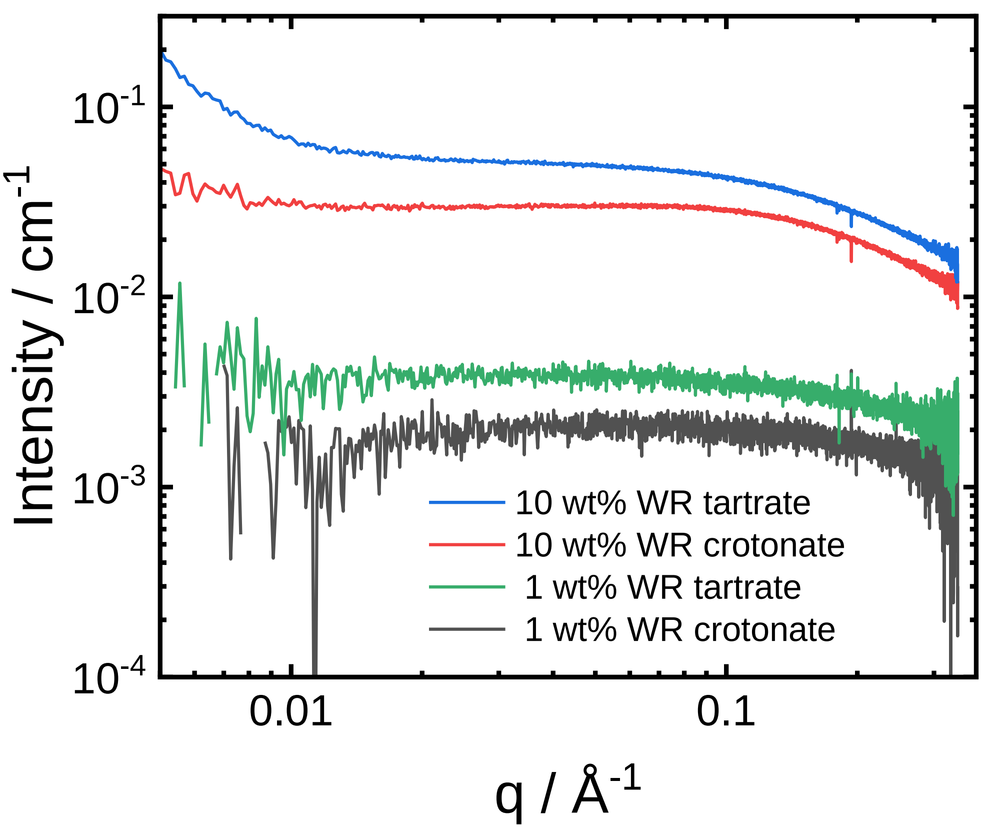

SI Fig.S5. SAXS from aqueous solutions of WR peptide, as indicated, including 10 wt% peptide salt solutions that are precursors for glass formation.

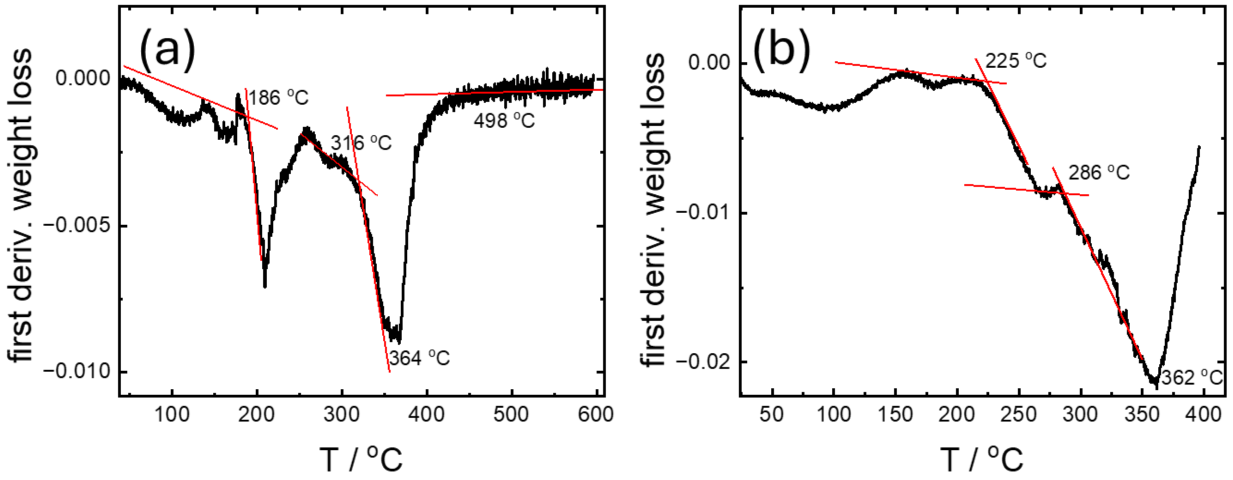

SI Fig.S6. First derivative of TGA data. (a) WR tartrate: 186 °C weight loss onset, 364 °C maximum decomposition temperature, 498 °C complete degradation, (b) WR crotonate: 225 °C weight loss onset, 362 °C maximum decomposition temperature.

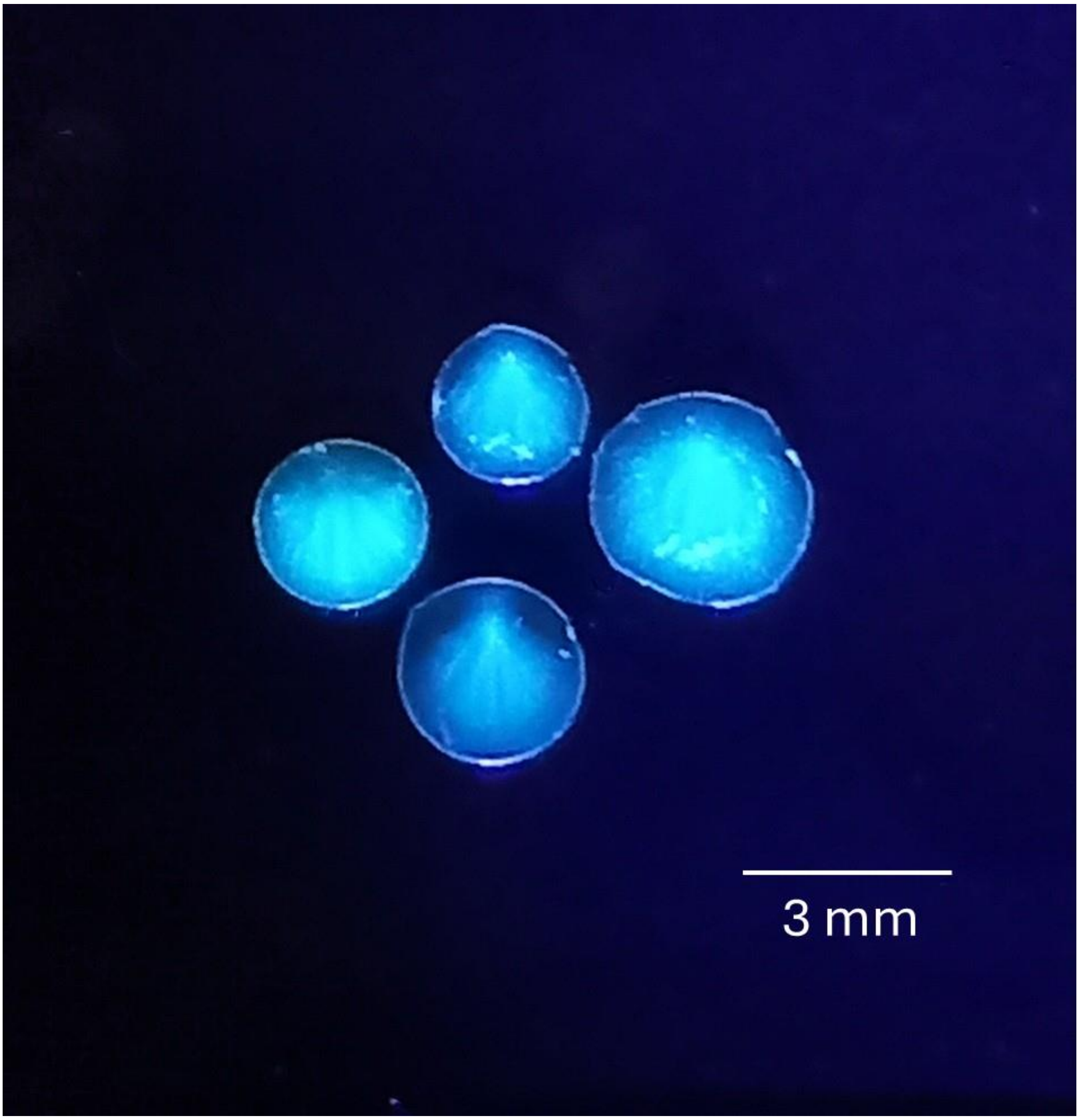

SI Fig.S7. Additional image showing fluorescence of WR crotonate glasses produced using a moulded patterned glass, similar to sample displayed in Fig. S1.

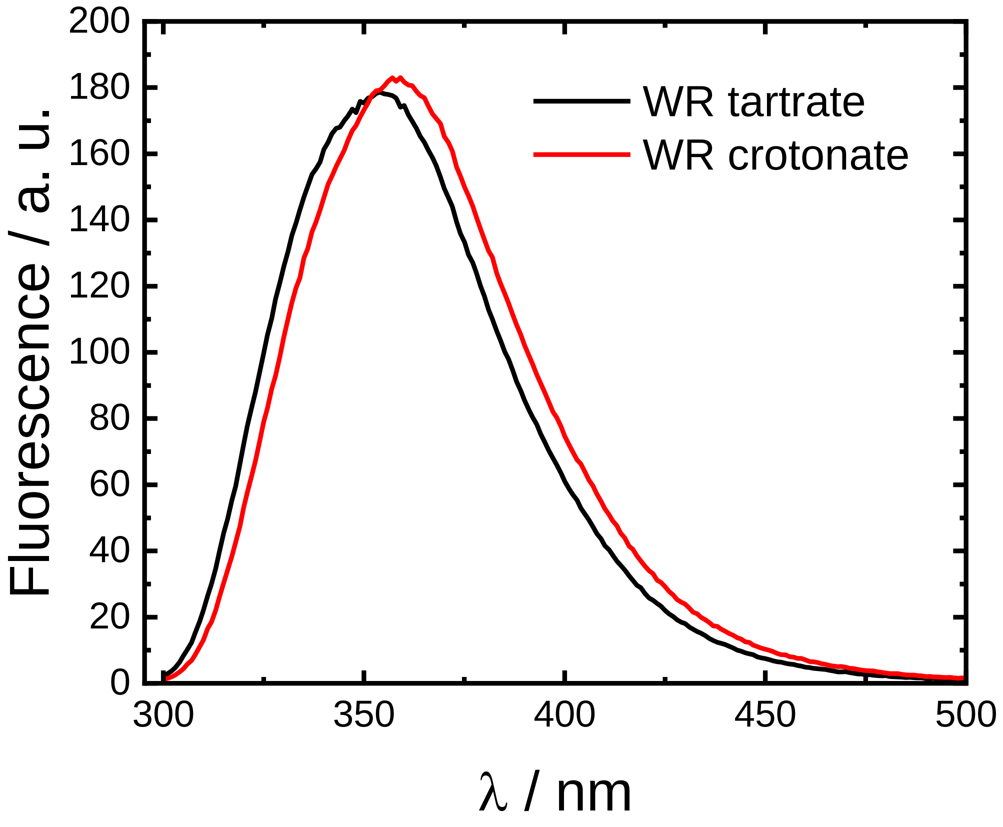

SI Fig.S8. Fluorescence emission spectra for solutions of WR glasses as indicated, prepared from 10 wt% solutions, upon excitation at λ = 280 nm.

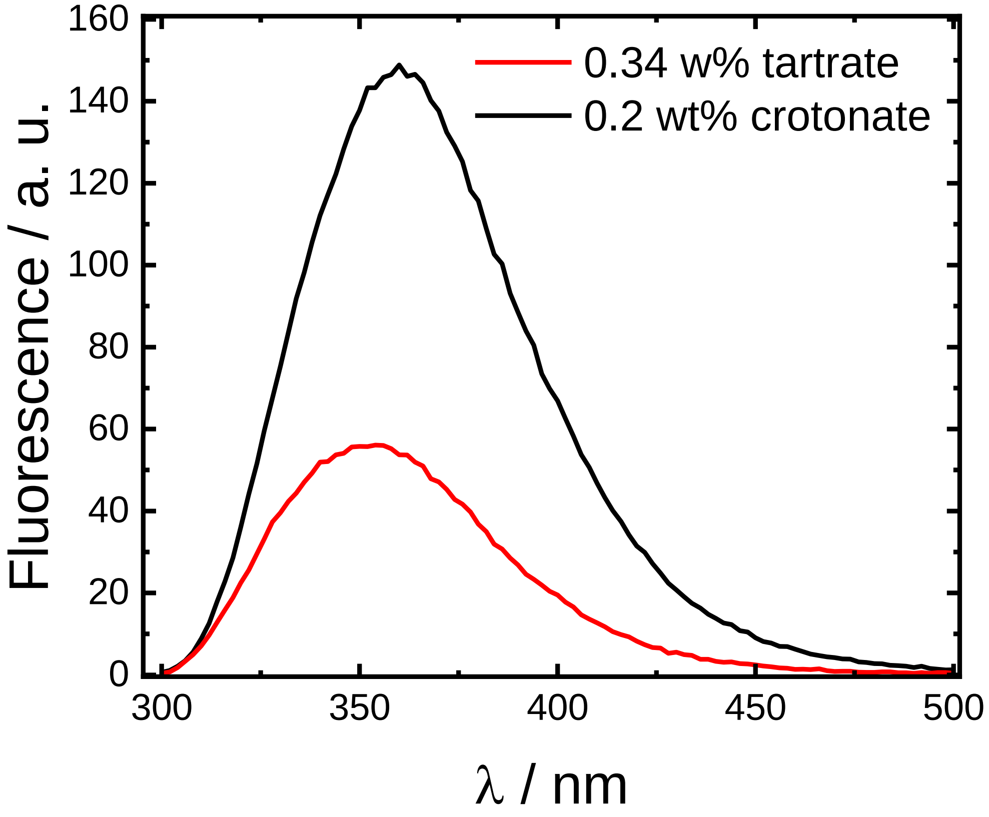

SI Fig.S9. Fluorescence emission spectra for solutions of WR salts as indicated upon excitation at λ = 280 nm.

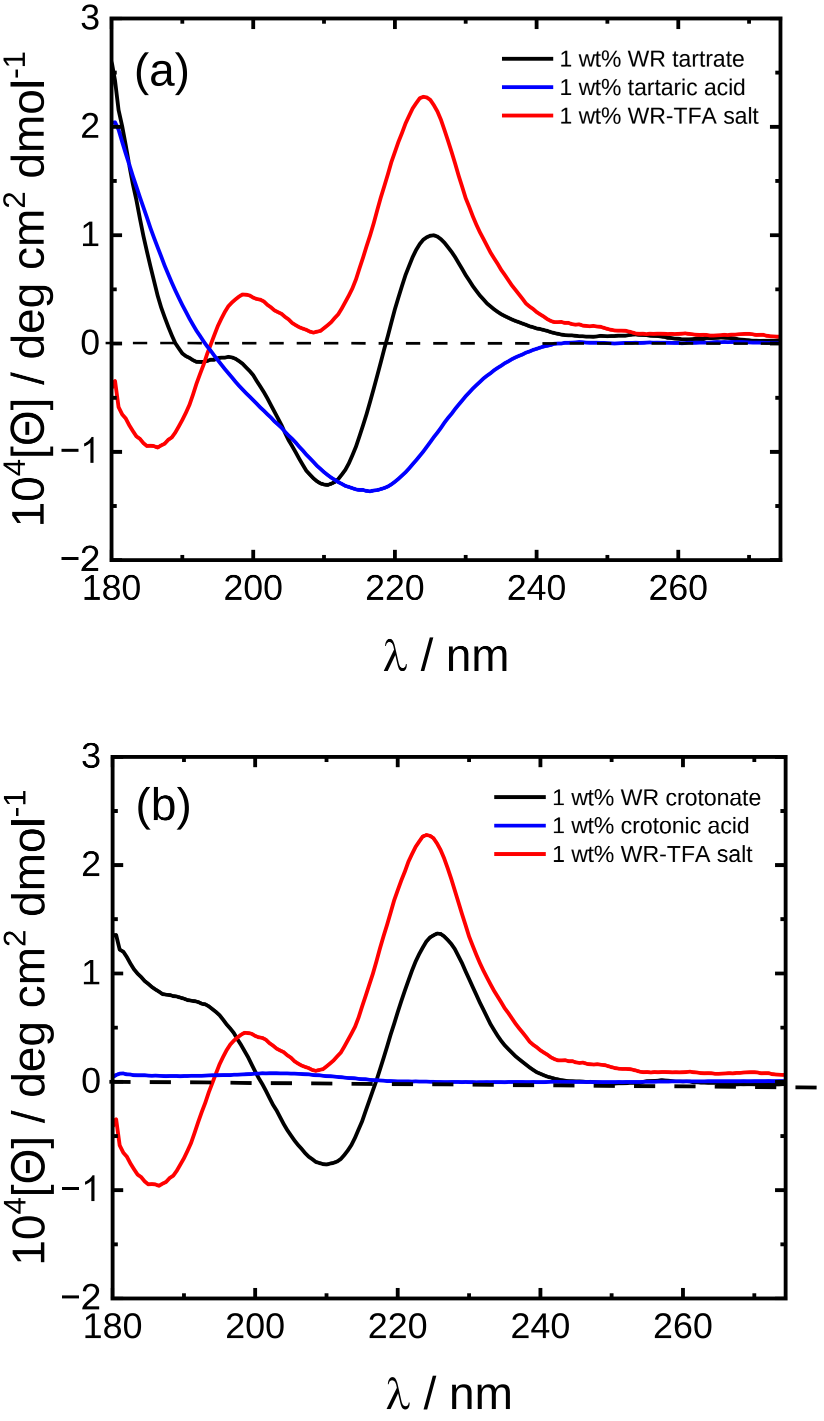

SI Fig.S10. CD spectra from 1 wt% peptide salt precursor solutions, 1 wt% organic salt solutions and 1 wt% WA-TFA salt solution, as indicated (TFA – trifluoroacetic acid). (a) WR tartrate, (b) WR crotonate. Data for WR-TFA is taken from ref.[1]

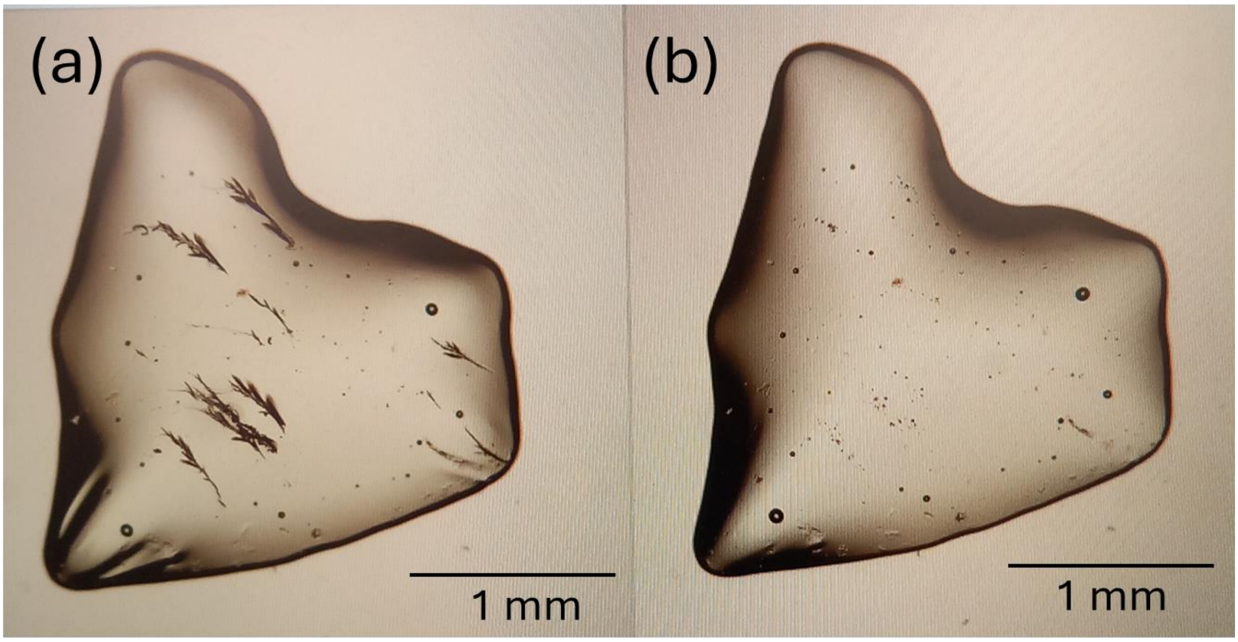

SI Fig.S11. Additional image of WR crotonate glass fragment. (a) Glass scratched with scalpel, (b) Glass after thermal treatment in humid atmosphere.

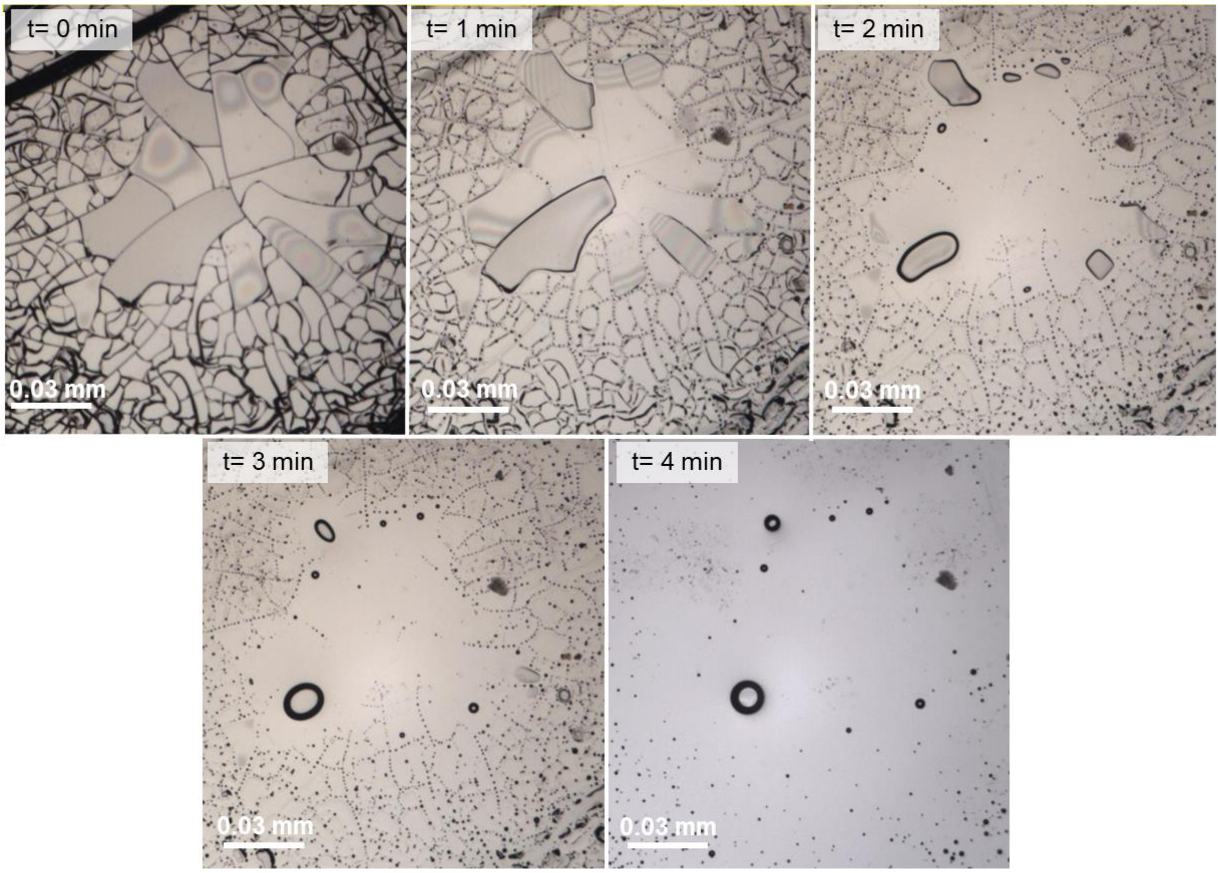

SI Fig.S12. Optical microscopy images showing self-healing under humid conditions of a cracked glass of WR tartrate at the time points indicated. Some bubbles were trapped in the healed glass evident in the lower images.

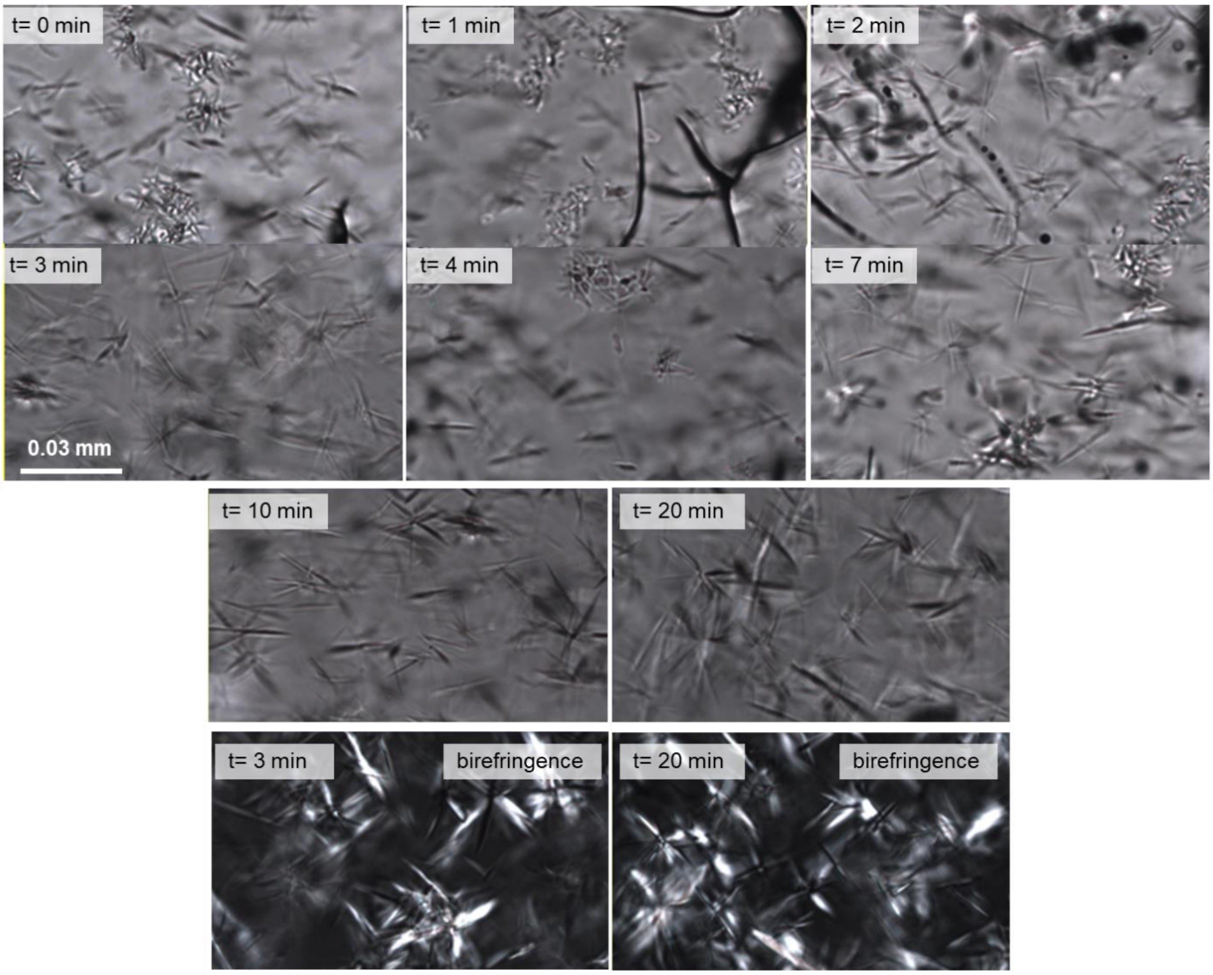

SI Fig.S13. Optical microscopy images (at a higher magnification than those in SI Fig. S12) for WR tartrate glass showing tartaric acid crystallites during the healing of a cracked glass of WR tartrate at the time points indicated. The lower two images were obtained using polarized optical microscopy.

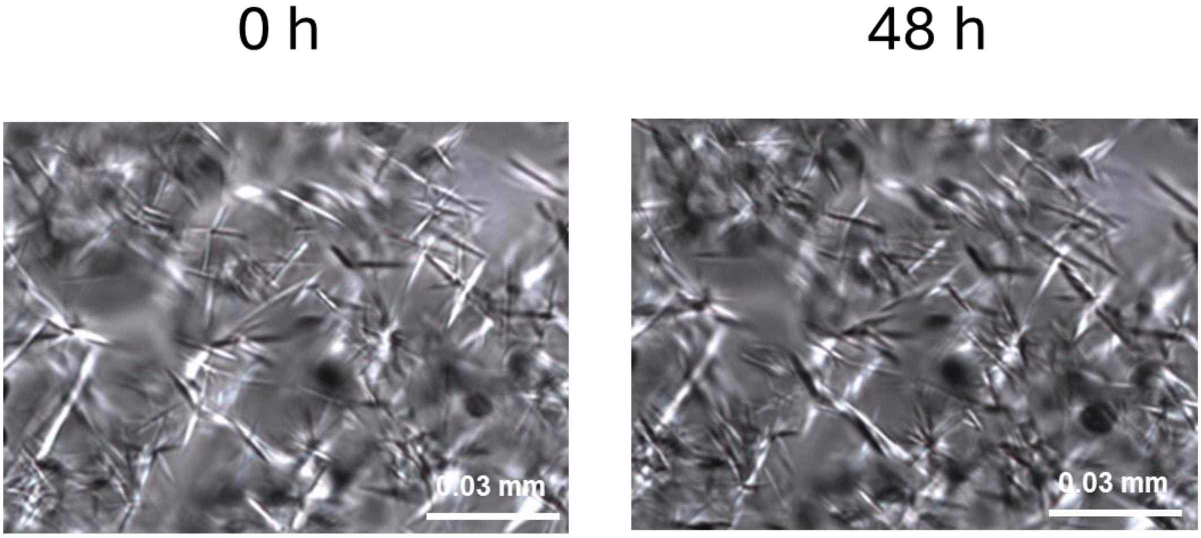

SI Fig.S14. Optical microscopy images for WR tartrate glass showing tartaric acid crystal-lites at room temperature immediately after (0 h) the thermal healing study (SI Fig.S12 and SI Fig.S13) and 48 h after.

SI Table S1. Selected Prior Work on Amino Acid and Peptide Glasses. T_g – glass transition temperature.

**Amino Acids/ Peptides**	$\mathbf{T_g}$	**Other Properties**	**Reference**
Range of Acetyl-, Fmoc-, and Cbz-capped amino acids	39 °C (Ac-Phe)	Wide-range optical transparency<br>Processing via 3D printing and moulding<br>Fabrication of colored and fluorescent glasses by doping with dyes etc<br>In vitro and in vivo biodegradability	[2]
His with hexamethylenetetramine	32-39 °C depending on solvent preparation	Wide-range optical transparency<br>High modulus<br>Tunable color ultralong room temperature phosphorescence	[3]
Cyclic dipeptides<br>Cyclo-Phe-Pro<br>Cyclo-Pro-Tyr<br>Cyclo-Trp-Tyr<br>Cyclo Trp-Trp	72 °C (Cyclo-Phe-Pro)	Wide-range optical transparency<br>High Modulus<br>Glasses from mixed cyclic peptides (including those which do not form glasses themselves) – so-called high entropy cyclic peptide glasses, with tunable mechanical properties	[4]
YYY	119 °C (after annealing and water loss)	Strong adhesion<br>Wide-range optical transparency<br>Self-healing	[5]
Fmoc-Leu and Fmoc-Leu with co-ordinated metal ions	37 °C (Fmoc-Leu)	Tunable colours and fluorescence of doped glasses<br>Ceramic-like mechanical properties (high modulus and hardness)	[6]
His, Lys and Arg with tartaric acid and other organic acids<br>Asp and Glu with organic bases	28 °C (His/tartaric acid)	Wide-range optical transparency<br>Tunable fluorescence color<br>Long afterglow glasses by blending with trimellitic acid phosphophore	[7]
Amino acids with organic acids<br>Peptides YY, PHF, thymopentin 5,	Amino acid or sequence dependent	Wide-range optical transparency<br>High refractive index<br>High modulus<br>Self-healing	[8]

tuftsin and others via polycarboxylic acid-mediated dehydration		Fabrication via 3D printing and other processing methods<br>Programmable biodegradation and recyclability	
D- or L-carnosine (β-Ala-His) with metal ions		Wide-range optical transparency<br>High refractive index<br>Self-healing<br>Highly circularly polarized luminescence	9

References

1. V. Castelletto, J. Seitsonen, A. Pollitt and I. W. Hamley, Minimal Peptide Sequences That Undergo Liquid-Liquid Phase Separation via Self-Coacervation or Complex Coacervation with ATP, *Biomacromolecules*, 2024, **25**, 5321–5331.
2. R. Xing, C. Yuan, W. Fan, X. Ren and X. Yan, Biomolecular glass with amino acid and peptide nanoarchitectonics, *Sci. Adv.*, 2023, **9**.
3. F. Nie and D. Yan, Macroscopic Assembly of Chiral Hydrogen-bonded Metal-free Supramolecular Glasses for Enhanced Color-tunable Ultralong Room Temperature Phosphorescence, *Angew. Chem., Int. Ed. Engl.*, 2023, **62**.
4. C. Yuan, W. Fan, P. Zhou, R. Xing, S. Cao and X. Yan, High-entropy non-covalent cyclic peptide glass, *Nature Nanotechnology*, 2024, **19**, 1840–1848.
5. G. Finkelstein-Zuta, Z. Arnon, T. Vijayakanth, O. Messer, O. Lusky, A. Wagner, G. Zilberman, R. Aizen, L. Michaeli, S. Rencus-Lazar, S. Gilead, S. Shankar, M. Pavan, D. Goldstein, S. Kutchinsky, T. Ellenbogen, B. Palmer, A. Goldbourt, M. Sokol and E. Gazit, A self-healing multispectral transparent adhesive peptide glass, *Nature*, 2024, **630**.
6. S. Cao, W. Fan, R. Chang, C. Yuan and X. Yan, Metal Ion-Coordinated Biomolecular Noncovalent Glass with Ceramic-like Mechanics, *CCS Chemistry*, 2024, **6**, 2814–2824.
7. X. Li, Y. Yang, Z. Zhao, S. Bai, Q. Li and J. Li, General and Versatile Nanoarchitectonics for Amino Acid-Based Glasses via Co-Assembly of Organic Counterions, *Angew. Chem., Int. Ed. Engl.*, 2025, **64**.
8. W. Fan, R. Xing, P. Zhou, G. Shen, S. Cao, C. Yuan and X. Yan, Programmable Bio-Derived Noncovalent Glass via Reconfigurable H-Bonding Networks, *Angew. Chem., Int. Ed. Engl.*, 2025, DOI: 10.1002/anie.202517982.
9. N. Li, L. Chang, Z. Gu and J. Zhang, Chiral Noncovalent Peptide Glasses for Highly Circularly Polarized Luminescence, *Adv. Mater.*, 2025, DOI: 10.1002/adma.202512857.
